# The efficacy of mouthwashes on oral microorganisms and gingivitis in patients undergoing orthodontic treatment: a systematic review and meta-analysis

**DOI:** 10.1186/s12903-023-02920-4

**Published:** 2023-04-06

**Authors:** Xiaolin Ren, Yuhan Zhang, Yong Xiang, Tao Hu, Ran Cheng, He Cai

**Affiliations:** Department of Preventive Dentistry, State Key Laboratory of Oral Diseases & National Clinical Research Center for Oral Diseases, West China Hospital of Stomatology, Sichuan University, 14#, 3Rd Section, Renmin South Road, Chengdu, Sichuan China

**Keywords:** Mouthwash, Orthodontic, Oral microorganisms, Gingivitis, Efficacy

## Abstract

**Background:**

Mouthwashes were convenient adjuncts to mechanical cleaning procedures. This review aimed to evaluate the efficacy of mouthwashes on oral microorganisms and gingivitis in orthodontic patients.

**Methods:**

By April 16, 2022, multiple databases and grey literature were searched based on the PICOS strategy. Randomized controlled trials in orthodontic patients evaluating the efficacy of mouthwashes with at least one microbial parameter and/or plaque- and/or gingival inflammation-related index were included. Relevant data were extracted, and the risk of bias was evaluated using Cochrane's tool. Individual mean and standard deviation of the outcomes in mouthwashes and placebos/blank controls were pooled to estimate the weighted mean differences (WMDs) and 95% confidence intervals (95%CIs). Sensitivity analysis, and certainty of evidence were evaluated.

**Results:**

Of 1684 articles, 32 studies satisfied the eligibility criteria, and nine were included for meta-analysis. Missing outcome data was the primary source of bias. Compared to blank controls, the short-term application of fluoride mouthwashes significantly reduced the colony counts of *Mutans streptococci (MS)*, while the long-term application may not be effective. Compared to placebos or blank controls, Chlorhexidine mouthwashes significantly reduced the colony counts of multiple microorganisms in the short-term. Compared to placebos or blank controls, herbal mouthwashes showed the inhibitory effect of *MS* in the short-term, with some results lacking statistical significance. After meta-analysis, significant lower plaque- and gingival inflammation-related indexes were observed in the Chlorhexidine mouthwashes groups [Gingival Index: WMD = -0.45, 95%CI = -0.70 to -0.20 (placebos as control); WMD = -0.54, 95%CI = -0.96 to -0.13 (blank controls); Plaque Index: WMD = -0.70, 95%CI = -1.12 to -0.27 (blank controls)]. Significant lower gingival inflammation-related indexes were observed in the herbal mouthwashes groups [Gingival Index: WMD = -0.20, 95%CI = -0.32 to -0.09 (blank controls)].

**Conclusions:**

The short-term application of fluoride mouthwashes may reduce the colony counts of cariogenic bacteria, but the long-term effect is not evident. Chlorhexidine may reduce the colony counts of multiple microorganisms in the short-term. Short-term application Chlorhexidine and herbal mouthwashes may effectively reduce plaque- and gingival inflammation-related indexes. However, the risk of bias, inconsistency, and imprecision in the included studies may reduce the certainty of the evidence.

**Supplementary Information:**

The online version contains supplementary material available at 10.1186/s12903-023-02920-4.

## Introduction

Orthodontic treatment aims to correct malocclusion and promote oral health, and periodontal health is vital to achieving this goal. However, orthodontic patients usually experience considerable difficulty in attaining appropriate oral hygiene. For instance, the gingival areas and the areas behind the archwire are prone to dental plaque accumulation, especially in children and patients who lack self-motivation [1]. Besides, orthodontic appliances increase the retention sites and complicate the process of efficient oral care procedures [2, 3]. Combining those factors in the orthodontic process usually leads to higher dental plaque retention and gingival inflammation [4]. Apart from the amount of dental plaque, the microbial composition may also change significantly during the orthodontic treatment [5]. There are alterations of the oral microbiota during orthodontic treatment, including increased *Streptococcus mutans (S. mutans)*, *Lactobacilli (LB)*, *Porphyromonas gingivalis* (*P. gingivalis*), and other potentially pathogenic gram-negative bacteria [6, 7].

Mechanical removal of dental plaque by toothbrushing, flossing, and using interdental brushes are common methods to maintain oral hygiene [8, 9]. Mouthwashes can be used as adjuncts to mechanical cleaning procedures, due to their ability to reach almost all residual dental plaque and ease of use [10]. The oral health-related ingredients in mouthwashes could be mainly classified as fluoride compounds, anti-microbial agents, or plant extracts [11]. Chlorhexidine (CHX), cetylpyridinium chloride (CPC), triclosan-copolymer, and essential oils are regarded as the most effective anti-microbial agents. They are prevalent ingredients in mouthwashes, exhibiting the ability to relieve gingival inflammation [12–14]. Moreover, essential oils contain complex natural mixtures, including terpenes and terpenoids, aromatic and aliphatic constituents [15], showing antioxidant activities [16, 17].

The anti-gingivitis and anti-microbial efficacy of numerous types of mouthwash during orthodontic therapy have been investigated, while controversial results existed [18–21]. Current systematic reviews mainly focused on certain types of mouthwash, showing that chlorhexidine mouthwash, essential oil mouthwash, and organic mouthwash effectively controlled dental plaque and gingival inflammation [22–24]. However, microbial changes caused by mouthwashes in orthodontic patients have not been fully assessed yet. There is evidence showing that repeated use of anti-microbial mouthwashes could alter the composition and metabolite profiles of the microbial community toward disease-associated traits and even lead to the development of antiseptic-resistant phenotypes [25, 26]. Thus, caution is required before recommending the use of generic anti-microbial products [27].

The study aimed to evaluate the efficacy of multiple types of mouthwash on oral microorganisms and gingivitis as adjuncts to mechanical cleaning procedures in orthodontic patients and to provide relevant evidence for clinical decision-making. The null hypothesis is that mouthwashes do not affect oral microorganisms and gingivitis symptoms in orthodontic patients.

## Methods

A protocol had been prepared in advance and pre-registered in the PROSPERO database (ID CRD42019127080). This systematic review was conducted in accordance with the Cochrane Handbook for Systematic Reviews and was reported according to the Preferred Reporting Items for Systematic Reviews and Meta-Analyses (PRISMA) 2020 statement [28].

### Participant-intervention-comparison-outcomes-study design (PICOS) question

The PICOS question of this systematic review could be clarified as *“What is the* efficacy *of mouthwashes on oral microorganisms and gingivitis compared to placebos or blank controls in orthodontic patients?”.*

### Eligibility criteria

Studies that met all the following criteria were included in this systematic review:

• Studies were designed as randomized controlled trials (RCTs).
• Studies were conducted on orthodontic patients of any age.
• The intervention group(s) should use mouthwash(es) as adjuncts to mechanical cleaning procedures (*e.g.,* toothbrushing) daily.
• The comparison group(s) should include placebo(s) or blank control(s) (before-after comparisons).
• Study outcomes should include the efficacy of mouthwashes on oral microorganisms and/or gingivitis, which contains at least one microbial parameter and/or plaque- and/or gingival inflammation-related index.
• Studies published in English or Chinese.

Studies combining mouthwashes with other positive interventions (*e.g*., electric toothbrushes), while the effect of mouthwashes cannot be distinguished by comparison with the control; retrospective cohort studies, *in-vitro* studies, animal or cadaver studies, case reports, reviews, letters, and editorials were excluded.

### Search strategy

By April 16, 2022, a literature search was conducted in the following databases: PubMed, EMBASE, and Cochrane Central Register of Controlled Trials (CENTRAL). The search strategy was based on the PICO-style process without filters or limitations of publication year, and the detailed process is shown in Table 1. To identify relevant publications thoroughly, ClinicalTrials.gov and the International Clinical Trials Registry Platform (ICTRP) were searched to identify unpublished clinical trials. The Conference Proceedings Citation Index-Science (CPCI-S) was searched via the Web of Science. In addition, the ProQuest Dissertations and Theses (PQDT) database was searched for dissertation and thesis. The GreyNet was also searched for any additional grey literature.

**Table 1:** The details of search strategy

Step	Search strategy
#1	Orthodontics.sh. OR orthodontic*
#2	(Mouthwashes.sh. OR mouthrinse* OR mouthwash* OR ((collut* OR gargle* OR rinse* OR wash*) AND (mouth* OR oral* OR dent*)
#3	Controlled Clinical Trial.pt. OR Controlled Clinical Trials as Topic.sh. OR Random Allocation.sh. OR random* OR Control Groups.sh. OR control* OR Placebos.sh. OR placebo*
#4	Review.pt. OR Case Reports.pt. OR Systematic Review.pt. OR Editorial.pt. OR Comment.pt. OR Models, Animal.sh. OR review.ti. OR systematic review.ti. OR case report.ti. OR case study.ti. OR case series.ti. OR animal model.ti. OR animal models.ti. OR mouse.ti. OR mouses.ti. OR rat.ti. OR rats.ti. OR mice.ti. OR rabbit.ti. OR rabbits.ti. OR rodent.ti. OR rodents.ti. OR beagle.ti. OR beagles.ti. OR dog.ti. OR dogs.ti. OR fish.ti. OR fishes.ti. OR swine.ti. OR swines.ti. OR pig.ti. OR pigs.ti
#5	#1 AND #2 AND #3 NOT #4

Abbreviations*: sh* MeSH subject headings; * Truncation symbol; pt, publication type; ti, title

### Study selection

Two independent reviewers (XLR and YX) utilized the Rayyan web application [29] to read the titles and abstracts of the literature and performed the preliminary screening. For better reliability, the reviewers were trained and standardized beforehand, and a substantial agreement threshold (> 0.81) [30] was reached by quantifying with Cohen's kappa coefficient (κ). During the title and abstract selection phase, an agreement rate of 97.6% was achieved between the two reviewers. Subsequently, full texts of relevant studies were obtained and independently screened based on the eligibility criteria by two reviewers. Any conflicting results were consulted with a third reviewer (HC).

### Data collection and outcome measurement

Two reviewers (XLR and YX) independently extracted relevant data in a well-designed data extraction sheet. The following study characteristics were extracted: authors and publication year, sample size, gender, age range, application of mouthwash(es) and comparison(s), relevant clinical measures, clinical effects, time of follow-up, loss to follow-up, and side effects. The changes in microorganisms were assessed by culture-based method, Polymerase Chain Reaction (PCR)-based method, metataxonomic, or metagenomic approaches. Gingivitis was evaluated using plaque- and/or gingival inflammation-related indexes (*e.g.,* plaque index and/or gingival index). Results at the follow-up endpoint were collected in studies with various observation time points.

### Risk of bias assessment

Two independent reviewers (XLR and YX) assessed the risk of bias of included studies using the revised Cochrane tool for RCTs (RoB2 tool) [31], with consultation from a third reviewer (HC) in case of any disagreement results. The tool evaluated five domains, including bias arising from the randomization process, bias arising from deviations from the intended interventions, bias due to missing outcome data, bias in the measurement of the outcome, and bias in the selection of the reported result, were assessed. The overall risk-of-bias judgment was based on the least favorable assessment. Publication bias was detected when there were 10 or more studies in meta-analysis.

### Synthesis process

Individual means and standard deviation (SD) of the same outcomes reported in relevant studies (*n* ≥ 3) comparing mouthwashes and placebos or blank controls (before-after comparisons) were pooled using RevMan (The Nordic Cochrane Centre, Copenhagen, Denmark. Version 5.4). The WMDs and 95%CIs were estimated between the two groups to evaluate the overall effect of mouthwashes. The heterogeneity of studies was quantified using the I-squared (*I*^*2*^) statistic, and the random-effect model was applied instead of the fixed-effect model when *I*^*2*^ was considerable (> 50%).

### Certainty of evidence

The Recommendation, Assessment, Development and Evaluation (GRADE) approach was used to investigate the quality of evidence with the online GRADEpro tool. Two independent reviewers (XLR and YX) evaluated the levels of certainty for outcomes based on five domains (risk of bias, inconsistency, indirectness, imprecision, and publication bias).

### Additional analysis

To determine the stability of the results, we investigated the influence of an individual study on the effect of the overall estimate by omitting one study in each turn using Stata software (Stata Corporation, Texas, USA. Version 16). We explored possible sources of heterogeneity by subgroup analysis (number of relevant studies ≥ 3) or meta-regression (number of relevant studies > 10).

## Results

### Study selection

**Fig. 1:**
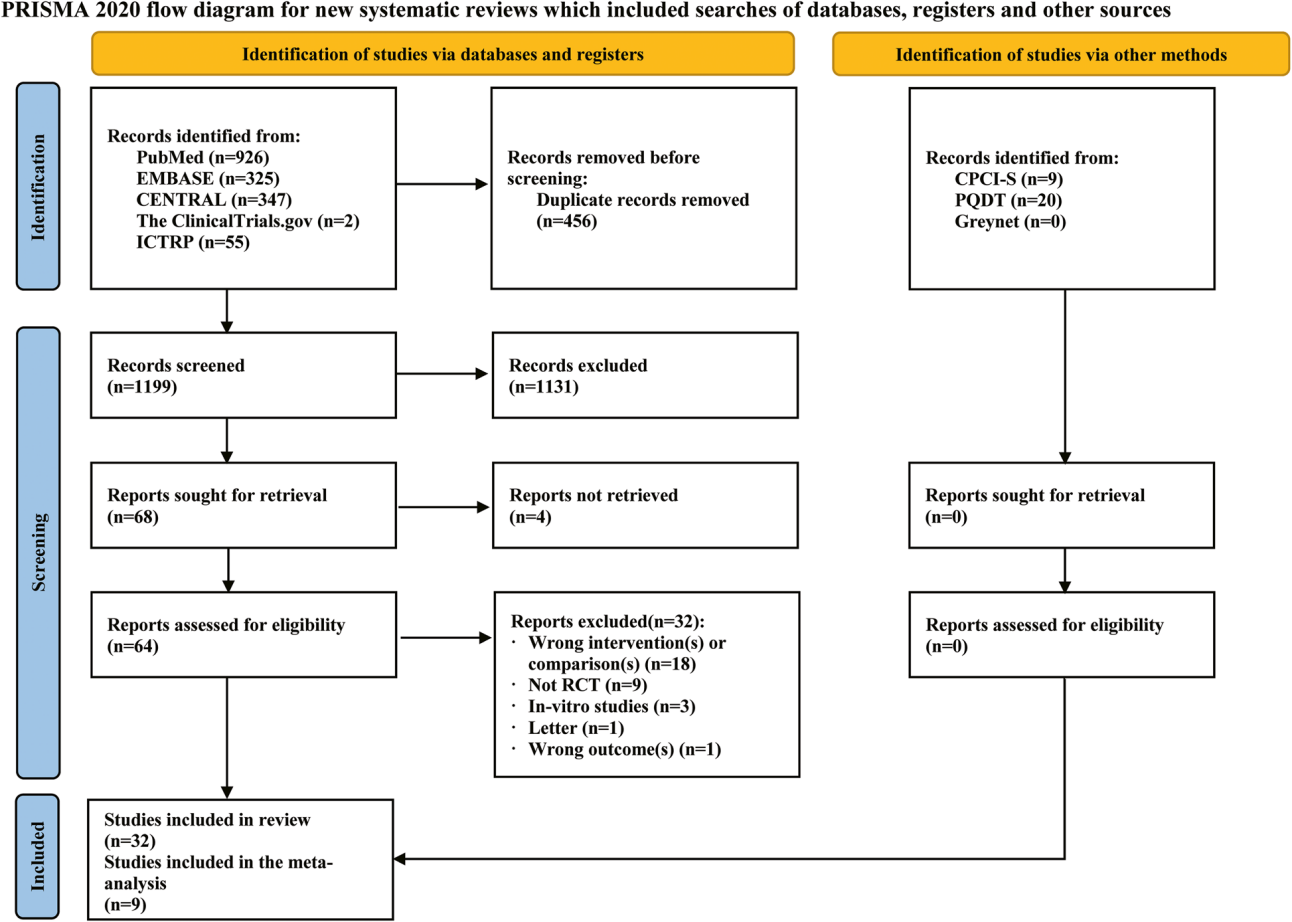
Preferred Reporting Items for Systematic Reviews and Meta-Analyses (PRISMA) flow diagram. CENTRAL, Cochrane Central Register of Controlled Trials; ICTRP, International Clinical Trials Registry Platform; CPCI-S, The Conference Proceedings Citation Index-Science; PQDT, The ProQuest Dissertations and Theses

This search strategy yielded a total of 1684 relevant articles. After removing duplicate items, 1228 studies remained. Furthermore, 1160 studies were excluded after screening the titles and/or abstracts. The full texts of the remaining 68 articles were sought for retrieval, and 32 articles were included in the current review as they fulfilled the eligibility criteria [18–21, 32–59], and nine articles were included for meta-analysis [20, 32, 35, 39, 49, 53, 55, 56, 58] (Fig. 1). The reason for exclusion is summarized in Additional file 1.

### General characteristics of included studies

The detailed characteristics of included studies are summarized in Additional file 2. The number of participants ranged from 25 to 270 in the included studies, with a total of 2007 participants. Most of the included studies were performed among young adults and adolescents, whereas eight studies recruited child participants below 12 years old [20, 21, 32, 41, 44, 50, 57, 59]. Most participants underwent fixed orthodontic treatment, while two studies included participants with removable orthodontic appliances and functional appliances [21, 59]. The follow-up time for most studies was short-term, which mainly ranged from four days to three months. The applications of fluoride mouthwashes were usually longer, with most studies following up to six months [42], a year [37, 59], or covering the whole orthodontic procedure [44, 57].

Several studies evaluated the microbial parameters. Among them, two studies quantitively assessed the level of red-complex bacteria in dental plaque through PCR [42, 43]. One study analyzed the changes in the microbiota composition through the 16 s rRNA gene sequencing technique [44]. Others assessed the level of specific microbial colonies after incubation of samples from saliva [18, 37, 38, 40, 48, 53, 59], dental plaque [33, 34, 36, 38, 42, 44], or orthodontic accessories [21, 43, 51, 52]. For gingivitis-related indexes, the Plaque Index (PI) by Silness and Löe [60] was the mainly adopted plaque-related index, and the Gingival Index (GI) by Löe and Silness [61] for gingival inflammation-related index. The majority of the studies were funded by government or non-profit entities, as well as industries. However, several studies did not disclose their funding sources. Detailed information is provided in Additional file 3.

### The efficacy of mouthwashes on microorganisms

Orthodontic treatment was significantly associated with changes in the microbial community [44], including increased colony counts of *Mutans streptococci (MS)*, *LB* [37], and higher alpha diversity of the microbiota [44]. The abundance of several other bacterial genera also showed significant differences. For instance, the abundance of health-associated genera *Streptococcus, Rothia*, and *Haemophilus* increased at the end and after the orthodontic treatment, whereas periodontal pathogens (such as *Selenomonas* and *Porphyromonas*) were most abundant during the orthodontic treatment [44]. Two short-term studies (within three months) indicated fluoride mouthwashes effectively reduced the colony counts of *MS* [33, 34], *LB* and total bacteria [34] compared to baseline. However, the long-term (over a year) application of fluoride mouthwash did not bring significant changes in *MS* and *LB* compared to blank controls [37, 59], and no significant difference in microbial composition was found compared to placebo [44]. Similarly, no inhibitory effect on the level of *P. gingivalis* was found in the fluoride mouthwash group at six months [42].

The most widely used mouthwash was CHX, although some studies conducted three-month trials with relatively lower concentrations of CHX (0.12%) [20, 32, 36], the application of CHX mouthwashes was mainly limited to four weeks in the included studies. Several studies consistently reported that CHX mouthwashes significantly reduced the colony counts of *MS* compared to placebos or blank controls [34, 38, 40, 48, 51–53]*.* Besides, inhibitory effects against colony forming units of *Streptococci* [21], total bacteria [34, 43], *LB* [34], and reduced relative quantification of red-complex bacteria (*P. gingivalis*, *Tannerella forsythia* (*T. forsythia)*, and *Treponema denticola* (*T. denticola*)) [43] were also reported. Furthermore, a better microbiologic change regarding the percentage of cocci, bacilli, and spirochetes was observed in dental plaque in the CHX mouthwash group compared to placebo [36]. However, CHX did not appear to alter the colony counts of *Candida albicans* (*C. albicans*) [40].

The application of herbal mouthwashes was also widespread, and most of them were limited to three months [19, 38–41, 45, 47, 48, 51, 52, 54, 56, 58]. Several studies investigated the efficacy of herbal mouthwashes on *MS*. Two studies showed that herbal mouthwashes significantly reduced colony counts of *MS* compared to placebo or blank control [48, 52], while others showed a short-time inhibitory effect [40] or an insignificant inhibitory trend [51]. Meanwhile, a six-month study showed that total aerobic and anaerobic bacteria, *Streptococci,* and *LB* [18] were not significantly altered in the herbal mouthwashes group. Similarly, it had no significant effect on *C. albicans* [40].

There were inconsistent results existed regarding the short-term effect of probiotic mouthwashes on colony counts of *MS* [33, 53]. Nevertheless, it significantly reduced the relative quantification of *P. gingivalis* at six months compared to baseline [42]. Besides, another short-term study revealed that the colony counts of total bacteria and relative quantification of red-complex bacteria were reduced in the chitosan mouthwash group. The best result was *T. denticola*, with a 58% reduction compared to baseline [43].

### The efficacy of mouthwashes on gingivitis

The majority of the studies affirmed that CHX mouthwashes were effective in reducing plaque- and gingival inflammation-related indexes compared to placebos or blank controls. Two studies evaluated the changes in pocket probing depth (PD), and it was significantly reduced in the CHX groups compared to the placebos [32, 56]. Some side effects were reported due to CHX application, such as tooth stain [20, 21, 32, 35, 48, 52], unpleasant flavor [35, 40, 52], burning sensation on the mucosa [32, 41, 47, 52], taste alteration [41, 47] and dry mouth [47], etc.

The herbal mouthwashes in the included studies mainly contained essential oils or plant extracts, such as Fructus mume, Salvadora persica, Zingiber officinale, Azadirachta indica, Aloe vera, Matricaria chamomilla, etc. Most studies indicated that herbal mouthwashes were effective in reducing plaque- and gingival inflammation-related indexes compared to placebos or blank controls, except for two studies using essential oils [18, 19] and Fructus mume mouthwash [18]. Herbal mouthwashes had a relatively low incidence of side effects compared to CHX [40, 47, 52]. However, it was worth noting that side effects such as unpleasant flavor, taste alteration, tooth stain, burning sensation, and dry mouth were also reported [40, 47, 52].

Regarding fluoride mouthwashes, a short-term study showed that compared to baseline or placebo, fluoride mouthwash significantly reduced the modified Quigley-Hein plaque index, modified gingival index, and bleeding index [34]. Other short-term studies indicated significantly reduced bleeding on probing, GI, and PI compared with baseline, while slightly lower relevant indexes than blank control [46]. Several long-term studies showed insignificant changes in relevant indexes compared to placebos or blank controls, indicating slightly lower bleeding scores and similar papillary bleeding index during the orthodontic procedure [57, 59], or slightly lower approximal plaque index (significant reduction only at six and nine months) and similar PI [37, 59].

Other types of mouthwashes were also applied. For instance, probiotic mouthwash [53] and chlorine dioxide mouthwash [58] were effective in reducing PI, modified plaque index, and GI compared to blank control. Furthermore, studies demonstrated that CPC mouthwashes were effective in reducing the bonded bracket plaque index [47], PI, plaque amount [38] and gingival bleeding index [50] compared to blank control. Regarding side effects, taste alteration, burning sensation, dry mouth, and unpleasant flavor were reported due to CPC mouthwash application [47].

### Risk of bias assessment

**Fig. 2:**
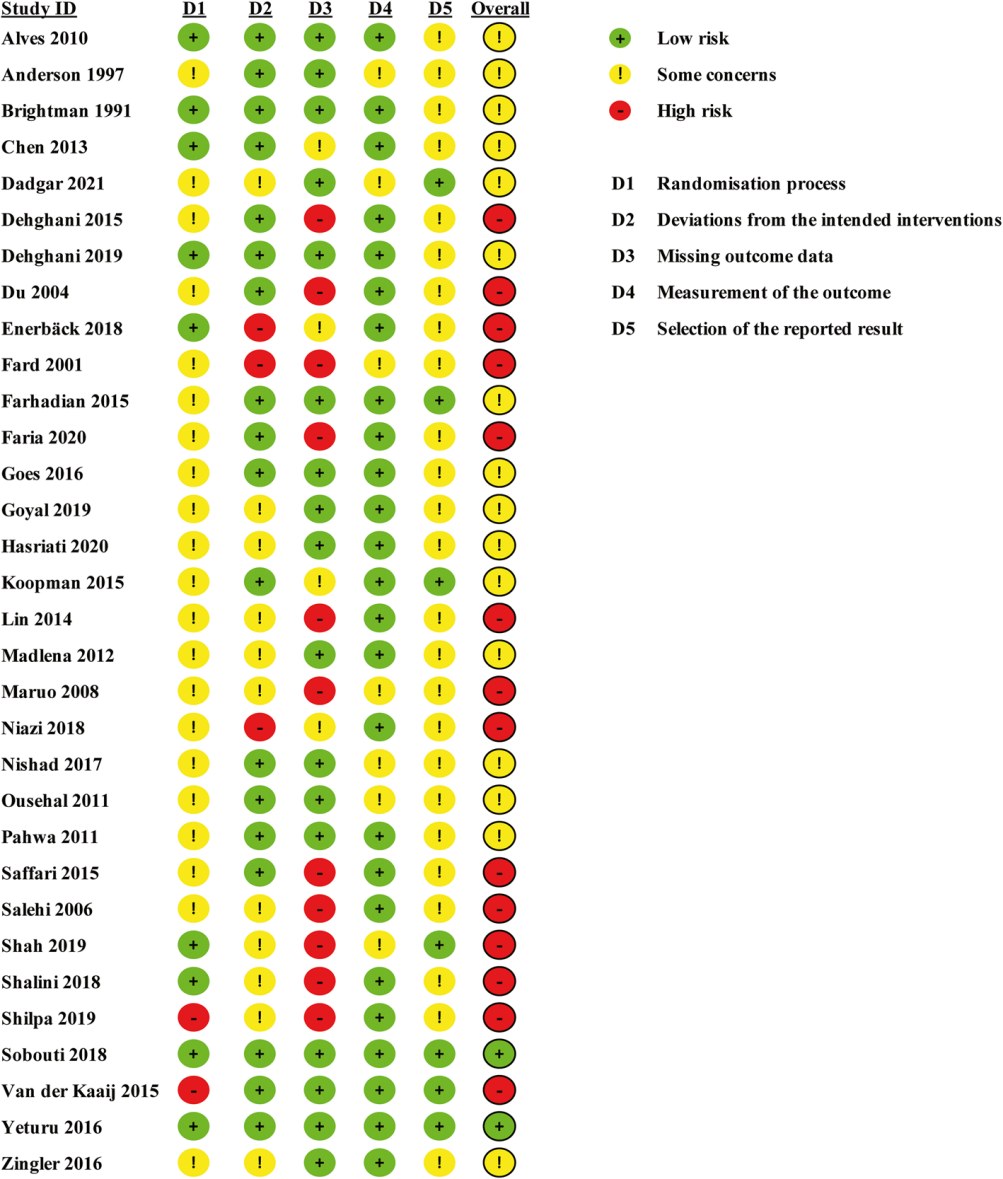
Review authors' judgments about each domain of bias across all included studies. Green represents a low risk of bias, yellow represents an unclear risk of bias, and red represents a high risk of bias

**Fig. 3:**
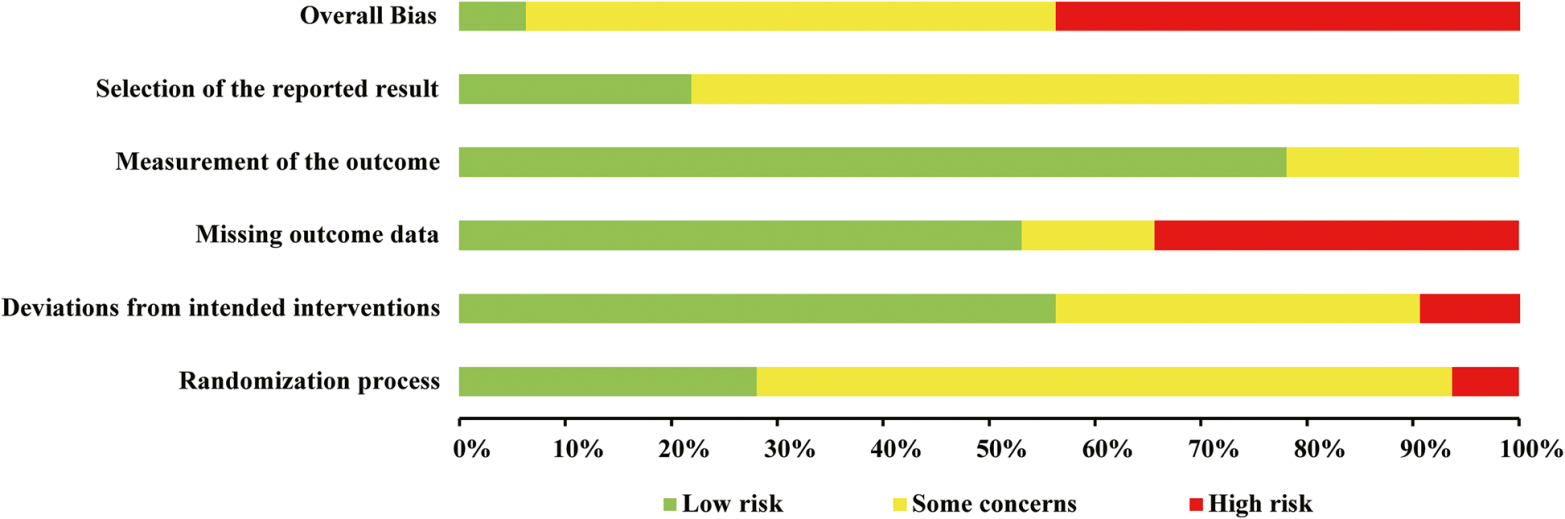
Review authors' judgments about the risk of bias presented as percentages across all included studies. Green represents a low risk of bias, yellow represents an unclear risk of bias, and red represents a high risk of bias

The risk of bias for each included study is shown in Fig. 2. The majority of studies had an uncertain or high risk of bias. Figure 3 displays the five domains and overall risk of bias in percentage form, where the missing outcome data was the primary source of bias, followed by deviation from intended intervention. Publication bias was not detected due to insufficient number of literatures in the meta-analysis.

### Meta-analysis results

A meta-analysis for microbial outcome is not feasible due to the lack of uniform units. For gingivitis outcome, when the CHX mouthwashes and placebos were compared through meta-analysis, three studies were included for GI [20, 32, 56]. When the CHX mouthwashes and blank controls were compared, six studies were included for the analysis of GI [35, 39, 49, 53, 55, 58] and four studies for PI [35, 49, 53, 55]. Regarding the comparison between herbal mouthwashes and blank controls, three studies were included for GI [35, 39, 58].

**Fig. 4:**
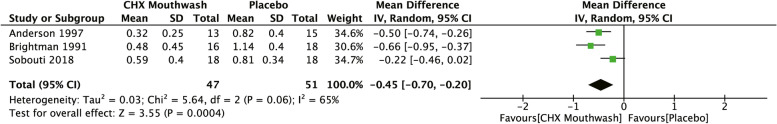
Forest plots comparing the efficacy between Chlorhexidine mouthwashes and placebos. Gingival Index (Löe and Silness 1963) was adopted

**Fig. 5:**
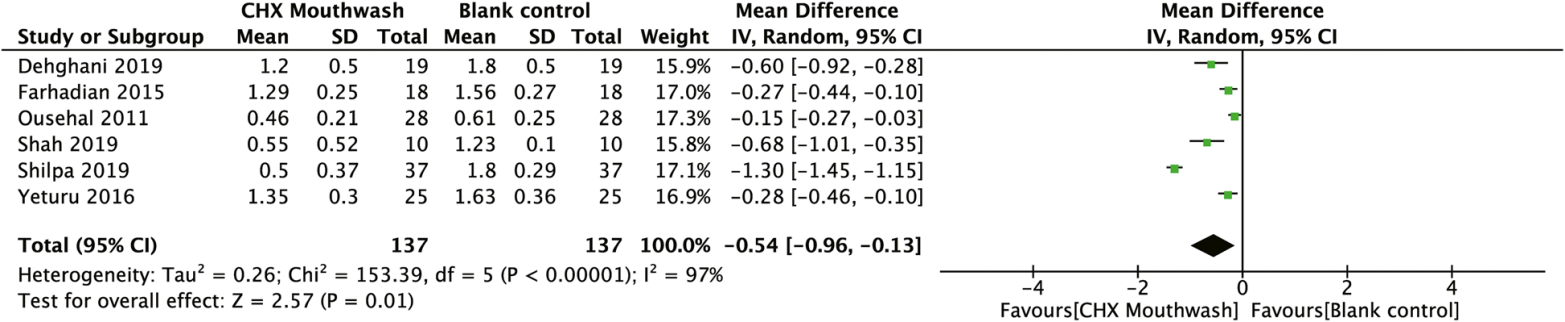
Forest plots comparing the efficacy between Chlorhexidine mouthwashes and blank controls. Gingival Index (Löe and Silness 1963) was adopted

**Fig. 6:**
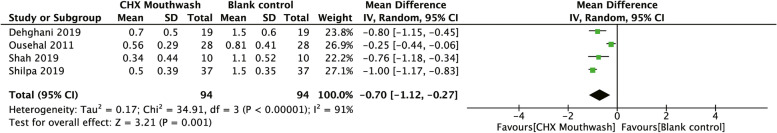
Forest plots comparing the efficacy between Chlorhexidine mouthwashes and blank controls. Plaque Index (Silness and Löe 1964) was adopted

**Fig. 7:**
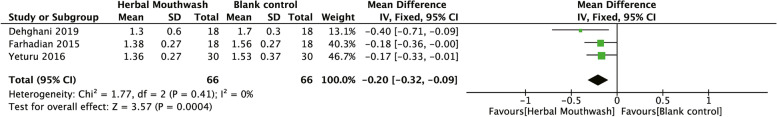
Forest plots comparing the efficacy between herbal mouthwashes and blank controls. Gingival Index (Löe and Silness 1963) was adopted

After meta-analysis, we graphically interpreted the synthesis data in the forest plots (Figs. 4–7). The mean GI scores of the CHX mouthwashes groups were significantly lower compared to placebos (WMD = -0.45, 95%CI = -0.70 to -0.20, *P* < 0.001, Fig. 4). Furthermore, the mean GI scores were significantly reduced in the CHX mouthwashes groups compared to the blank controls (WMD = -0.54, 95%CI = -0.96 to -0.13, *P* = 0.01, Fig. 5).

Similarly, significantly lower PI scores were observed in the CHX mouthwashes groups compared to the blank controls (WMD = -0.70, 95%CI = -1.12 to -0.27, *P* = 0.001, Fig. 6). Significantly lower GI scores were observed in the herbal mouthwashes groups compared to the blank controls (WMD = -0.20, 95%CI = -0.32 to -0.09,* P* < 0.001, Fig. 7). Moreover, the participants in the meta-analysis studies exhibited mild to moderate gingival inflammation at baseline with mean GI scores of 0 to 2.0.

### Certainty of evidence

The certainty of evidence was determined as moderate to very low, with most comparisons as low or very low (Table 2). The main reasons for downgrading the certainty of the evidence were inconsistency between studies, imprecision due to the small sample size, and the high risk of bias in the included studies.

**Table 2:** Summary of main findings by the Grading of Recommendations Assessment, Development, and Evaluation (GRADE)

Type of MW	Certainty assessment						Effect	
	Number of studies	Risk of bias	Inconsistency	Indirectness	Imprecision	Other considerations	Absolute (95% CI) OR qualitative assessment	Certainty
CHX MW	Cariogenic bacteria— MW vs placebos 4 RCT (≤ 3 month)	serious^a^	serious^b^	not serious	serious^c^	publication bias undetected	Colony count of *MS* was significantly reduced compared to placebos according to three studies [34, 48, 52] Colony count of *MS* was slightly reduced compared to placebo in a short time (significantly at 15 min) [40]	⨁◯◯◯ Very low
	Cariogenic bacteria— MW vs blank controls 2 RCT (≤ 3 month)	very serious^d^	serious^b^	not serious	very serious^e^	publication bias undetected	Colony count of *MS* was significantly reduced compared to blank control according to one study [53] Colony count of *MS* was significantly reduced compared to baseline according to one study [51]	⨁◯◯◯ Very low
	Total bacteria— MW vs blank controls 2 RCT (≤ 3 month)	serious^a^	serious^b^	not serious	very serious^e^	publication bias undetected	Colony count of total bacteria were significantly reduced compared to placebos according to two studies [34, 43]	⨁◯◯◯ Very low
	GI—MW vs placebos 3 RCT (≤ 3 month)	not serious	serious^f^	not serious	serious^c^	publication bias undetected	WMD 0.45 lower (0.70 lower to 0.20 lower) [20, 32, 56]	⨁⨁◯◯ Low
	GI—MW vs blank controls 6 RCT (≤ 3 month)	serious^g^	serious^f^	not serious	serious^c^	publication bias undetected	WMD 0.54 lower (0.96 lower to 0.13 lower) [35, 39, 49, 53, 55, 58]	⨁◯◯◯ Very low
	PI—MW vs placebos 2 RCT (≤ 3 month)	serious^g^	not serious	not serious	very serious^e^	publication bias undetected	PI was significantly reduced compared to placebos according to two studies [20, 32]	⨁◯◯◯ Very low
	PI—MW vs blank controls 4 RCT (≤ 3 month)	serious^a^	serious^f^	not serious	serious^c^	publication bias undetected	WMD 0.7 lower (1.12 lower to 0.27 lower) [35, 49, 53, 55]	⨁◯◯◯ Very low
	PD—MW vs placebos 2 RCT (≤ 3 month)	not serious	not serious	not serious	very serious^e^	publication bias undetected	PD was significantly reduced except mid-lingual site compared to placebo [32]. The changes of PD were significantly higher compared to placebo [56]	⨁⨁◯◯ Low
Herbal MW	Cariogenic bacteria— MW vs placebos 2 RCT (≤ 3 month)	very serious^d^	serious^b^	not serious	very serious^e^	publication bias undetected	Colony count of *MS* was significantly reduced compared to placebo [52]. Colony count of *MS* was slightly reduced compared to placebo in a short time (significantly at 1 and 15 min) [40]	⨁◯◯◯ Very low
	Cariogenic bacteria— MW vs blank controls 2 RCT (≤ 3 month)	serious^a^	serious^b^	not serious	very serious^e^	publication bias undetected	Colony count of *MS* was significantly reduced compared to baseline [48]. Colony count of *MS* was insignificantly reduced compared to baseline [51]	⨁◯◯◯ Very low
	GI—MW vs blank controls 3 RCT (≤ 3 month)	not serious	not serious	not serious	serious^c^	publication bias undetected	WMD 0.2 lower (0.32 lower to 0.09 lower) [35, 39, 58]	⨁⨁⨁◯ Moderate
	Plaque related index— MW vs placebos 3 RCT (≤ 3 month)	not serious	serious^h^	not serious	serious^c^	publication bias undetected	PI’ was significantly reduced compared to placebo [56] VPI was significantly reduced compared to placebo [41] PI was insignificantly reduced compared to placebo [19]	⨁⨁◯◯ Low
	Plaque related index— MW vs blank controls 8 RCT (≤ 3 month)	serious^a^	serious^h^	not serious	serious^c^	publication bias undetected	PI’ was significantly reduced compared to baseline [39] PI’’ was significantly reduced compared to baseline [48] PI’’ was significantly reduced compared to baseline [54] PI’’’ was significantly reduced compared to baseline [58] BBI was significantly reduced compared to baseline [40] BBPI was significantly reduced compared to blank control [47] TI was significantly reduced compared to baseline [45] The amount of dental plaque was significantly reduced compared to baseline [38]	⨁◯◯◯ Very low
	Gingival inflammation related index—MW vs placebos 3 RCT (≤ 3 month)	serious^g^	serious^i^	not serious	serious^c^	publication bias undetected	GBI was significantly reduced compared to placebo [41] GBI’ was significantly reduced compared to placebo [56] BI was significantly reduced compared to placebo [40]	⨁◯◯◯ Very low
	Gingival inflammation related index—MW vs blank controls 3 RCT (≤ 3 month)	serious^a^	serious^i^	not serious	serious^c^	publication bias undetected	CPI was significantly reduced compared to baseline [35] SBI was significantly reduced, while MGI was similar compared to baseline [45] BOP was significantly reduced compared to blank control [39]	⨁◯◯◯ Very low
Fluoride MW	Cariogenic bacteria—MW vs blank controls 2 RCT (≤ 3 month)	serious^a^	serious^b^	not serious	very serious^e^	publication bias undetected	Colony count of *MS* was significantly reduced compared to baseline according to two studies [33, 34]	⨁◯◯◯ Very low
	Cariogenic bacteria—MW vs blank controls 2 RCT (≥ 12 month)	serious^a^	serious^b^	not serious	very serious^e^	publication bias undetected	Colony count of *MS* was similar to blank control according to two studies [37, 59]	⨁◯◯◯ Very low
	Cariogenic bacteria—MW vs blank controls 2 RCT (≥ 12 month)	serious^a^	serious^b^	not serious	very serious^e^	publication bias undetected	Colony count of *LB* was similar to blank control according to two studies [37, 59]	⨁◯◯◯ Very low
	Plaque related index— MW vs blank controls 2 RCT (≤ 3 month)	serious^a^	serious^h^	not serious	very serious^e^	publication bias undetected	TI was significantly reduced compared to baseline [34]. PI was significantly reduced compared to baseline, while similar with blank control [46]	⨁◯◯◯ Very low
	Plaque related index— MW vs blank controls 2 RCT (≥ 12 month)	serious^a^	serious^h^	not serious	very serious^e^	publication bias undetected	PI was similar to blank control [37] API was slightly reduced compared to blank control (significant at 6 and 9 months) [59]	⨁◯◯◯ Very low
	Gingival inflammation related index—MW vs blank controls 2 RCT (≤ 3 month)	serious^a^	serious^i^	not serious	very serious^e^	publication bias undetected	MGI’ and BI’ was significantly reduced compared to baseline [34]. BOP and GI was slightly lower compared to blank control [46]	⨁◯◯◯ Very low
CPC MW	Plaque related index— MW vs blank controls 3 RCT (≤ 3 month)	serious^a^	serious^h^	not serious	serious^c^	publication bias undetected	BBPI was significantly reduced compared to blank control [47] PI was significantly reduced compared to blank control [50] The amount of dental plaque was significantly reduced compared to baseline [38]	⨁◯◯◯ Very low
Probiotic MW	Cariogenic bacteria—MW vs blank controls 2 RCT (≤ 3 month)	serious^a^	serious^b^	not serious	very serious^e^	publication bias undetected	Colony count of *MS* was significantly reduced compared to blank control [53]. Colony count of *MS* was insignificantly increased compared to baseline [33]	⨁◯◯◯ Very low

The results reported in two or more studies are included in the table

Abbreviations: *CHX* Chlorhexidine, *CPC* Cetylpyridinium chloride, *MW* Mouthwash, *RCT* Randomized controlled trial, *CI* Confidence interval, *WMD* Weighted mean difference, *GI* Gingival index by Löe and Silness 1963, *PD* Pocket probing depth, *PI* Plaque index by Silness and Löe 1964, *MS Mutans streptococci*, *PI*’ Plaque index by O’leary’s 1972, *VPI* Visible plaque index, *PI*’’ Plaque index (Unspecified), *PI’’’* Plaque index by William 1991, BBI Bonded bracket index, *BBPI* Bonded bracket plaque index, *TI* Quigley-Hein plaque index, *GBI*, Gingival bleeding index by Ainamo J 1975, *GBI’* Gingival bleeding index by Carter and Barnes 1974, BI Bleeding index (Unspecified), *CPI* Community periodontal index, *SBI* Sulcus bleeding index, *BOP* Bleeding on probing, *MGI* Modified gingival index (Unspecified), *LB Lactobacilli*, *API* Approximal plaque index, *MGI’* Modified gingival index by Lobene 1986, *BI’* Bleeding index by Saxton and van der Ouderaa 1989

a. The proportion of information from studies at high risk of bias is sufficient to affect the interpretation of results and to lower confidence in the estimate of effect

b. Lack of uniform units

c. Sample sizes that are less than 400

d. The proportion of information from studies at high risk of bias is sufficient to affect the interpretation of results and to substantially lower confidence in the estimate of effect

e. The effect estimate came from only one or two studies with small sample sizes

f. Minimal overlap of CI and *I*^*2*^ > 50%

g. Most information is from studies at unclear risk of bias, and it is likely to lower confidence in the estimate of effect

h. Adopt different plaque-related indexes

i. Adopt different gingival inflammation-related indexes

### Additional analyses

The sensitivity analysis results remained robust for all meta-analysis results (Additional file 4). Due to the limited number of studies, subgroup analysis or meta-regression was not applicable.

## Discussion

To the best of our knowledge, systematic reviews evaluating the efficacy of multiple types of mouthwash on microorganisms and gingivitis in orthodontic patients are still lacking. The null hypothesis was rejected based on the current findings, as mouthwashes may effectively reduce multiple microorganisms and relieve gingivitis. Among the various types of mouthwash, there is evidence supporting the short-term application of fluoride mouthwash may inhibit the colony count of *MS*, while the long-term application may not alter its level. The short-term application of CHX and herbal mouthwashes may reduce the colony count of *MS* and relieved gingivitis. However, the certainty of the evidence of most comparisons are low or very low due to the risk of bias, inconsistency, and imprecision in the included studies, and these findings should be interpreted with caution.

Several studies conducted at least one-year follow-up and found orthodontic treatment is significantly associated with microbial changes [44, 59]. Fluoride mouthwashes were effective in reducing the colony counts of *MS* [33, 34] and *LB* [34] in the short-term (within three months), while its long-term (over a year) inhibitory effect was not as evident [37, 59]. *MS* is a group of critical etiologic agents for dental caries consisting of *S. mutans*, *Streptococcus sanguis*, and other species [62]. *MS* colonizes on the tooth surface by synthesizing large amounts of dextran extracellular polymers from sucrose [63]. In addition, the ability to metabolize various carbohydrates to form organic acids creates a favorable environment for the reproduction of other acidogenic species, *e.g., LB* [64].

High-throughput sequencing provides more details for analyzing potential microbiota changes compared to culture methods that aim at specific microbe [65]. A 16 s rRNA gene sequencing technique-based study found there was a trivial difference in the microbiota composition between fluoride mouthwash and the placebo group during the whole orthodontic procedure, which promoted the growth of neither health nor disease-associated bacteria [44]. However, long-term studies using culture-independent methods are still lacking for other types of mouthwashes.

In mouthwashes with fluoride as the main active ingredient, common compounds include sodium fluoride (NaF) and amine fluoride (AmF), and the total concentration of fluoride is usually around 230–250 ppm. It is worth noting that fluoride is a prevalent additive that is often combined with other ingredients to formulate mouthwashes with multiple active components (*e.g.*, CHX and NaF [34, 38, 56], CPC and NaF [38]). The addition of fluoride may increase the efficacy of mouthwashes. For instance, adding NaF enhanced the anti-*LB* effect of CHX at low concentrations (0.06%) [34]. The benefit of fluoride may be related to both promoting tooth mineralization and its anti-microbial effect [66]. While fluoride is effective in preventing caries, it is essential to pay attention to its concentrations. Over-the-counter mouthwashes containing 0.05% NaF (230 ppm fluoride) are available for daily rinsing for individuals above six years old, whereas 0.20% NaF (920 ppm fluoride) should be applied under controlled conditions weekly [67].

There were, however, conflicting results regarding the anti-gingivitis efficacy of fluoride mouthwashes. A short-term study found that plaque- and/or gingival inflammation-related indexes were significantly reduced compared to baseline in the fluoride mouthwash group [34]. Yet another short-term study lacks statistically significant improvement, suggesting slightly lower relevant indexes compared to the blank control in the fluoride mouthwash group [46]. And its long-term effect may also not significant [59]. As the duration of orthodontic treatment increases, gingivitis may become more advanced [57] and more difficult to control. Therefore, future studies aiming at different severity and follow-up times are still needed.

CHX is a widely used broad-spectrum anti-microbial agent [68, 69]. The included studies indicated that CHX mouthwashes significantly reduced colony counts of multiple cariogenic microorganisms *i.e.*, *MS* [34, 38, 40, 48, 51–53], *Streptococci* [21], and *LB* [34]. Besides, a study showed the CHX group reduced *P. gingivalis* count by 55.8%, 25.3% of the *T. forsythia* count, and 42.6% of the *T. denticola* count [43]. These bacteria are known as the red-complex and are considered a group of periodontal pathogens [70]. However, we are currently moving away from simply "killing" bacteria to a view of managing the oral microbiome [71]. CHX may bring a shift change in the diversity and abundance of the oral microbiome, and the role of such changes is not fully understood [72].

Compared to placebos or blank controls, most of the included studies and pooled effect estimates agree on the absolute anti-gingivitis efficacy of CHX. Despite the meta-analyses for GI (Fig. 5) and PI (Fig. 6) in CHX mouthwashes versus blank controls indicating very high heterogeneity, all trials agreed on the direction of the effect that benefits CHX mouthwashes and remained stable in the sensitivity analysis. Therefore, this mainly influenced the effect magnitude rather than the certainty of its effectiveness.

Four studies evaluated the effect of herbal mouthwashes on *MS*, showing the different magnitude of inhibition. Two studies found that Persica and Azadirachta indica mouthwashes significantly reduced colony counts of *MS* [48, 52]. In another study, Persica mouthwash also showed a tendency to inhibit *MS*, but not to a statistically significant level [51]. One study found Zingiber officinale essential oil mouthwash had a short-term inhibitory effect, the colony counts of *MS* reduced immediately after the mouthwash application, while the inhibitory effect diminished at seven days follow-up [40]. The effect of herbal mouthwashes on total aerobic and anaerobic bacteria, *Streptococci, LB* [18], and *C. albicans* [40] were also evaluated, while no evident inhibitory effects of these microorganisms were identified.

In line with most included studies, the pooled effect estimates also demonstrated that herbal mouthwashes effectively reduced gingival inflammation compared to blank controls or placebos. A meta-analysis for dental plaque outcomes is not feasible due to the lack of sufficient studies and uniform units. While some studies have suggested that there were no significant differences in dental plaque levels in the essential oils [18, 19] or Fructus mume mouthwash group [18] compared to blank control or placebo, extensive studies have demonstrated that herbal mouthwashes effectively reduce dental plaque. The different types of natural extracts in herbal mouthwashes and different follow-up times may contribute to the discrepancy.

There were limited or conflicting results regarding the effect of other types of mouthwashes on the microorganisms. Compared to the blank control, the colony counts of *MS* were significantly reduced in the *Lactobacillus*-based mouthwash group [53]. However, they were insignificantly increased in the *Lactobacillus plantarum*-based mouthwash group [33]. This difference may be related to the formulation of probiotics and the duration of mouthwash application [33]. Moreover, there was a significant decrease in *P. gingivalis* levels in the *Lactobacillus* and *Bifidobacterium* based-probiotic mouthwash group compared to baseline [42]. Only one study evaluated the anti-microbial efficacy of chitosan mouthwash, colony counts of total bacteria, and the relative quantification of red complex bacteria reduced compared to baseline. *T. denticola* showed the best suppression, with a 58% decrease, comparable to CHX [43].

A limited number of studies showed that probiotic mouthwash and chlorine dioxide mouthwash were effective in reducing plaque- and/or gingival inflammation-related indexes compared to blank controls, showing a similar effect to the CHX group [53, 58]. Besides, CPC mouthwashes also significantly reduced plaque- and/or gingival inflammation related indexes [38, 47, 50]. However, it did not appear to improve inflammation in the marginal gingiva, which could be related to its low concentration (0.07%) [50]. The anti-gingivitis effects of these mouthwashes are promising, and further research is still needed due to the limited number of studies available.

Interestingly, the tendency to improve plaque- and/or gingival inflammation-related indexes compared to baseline was also commonly observed in the placebo groups, reaching a statistically significant level in several studies [19, 20, 34, 40]. This phenomenon may be explained by the Hawthorne effect, which commonly exists and may lead to overestimated results [73]. On the other hand, rinsing with water after eating might help maintain oral hygiene by diluting bacterial chemical compounds and carbohydrate residues [56].

Regarding the safety of mouthwash, some side effects have been reported with CHX application, such as tooth stain [20, 21, 32, 35, 48, 52], unpleasant flavor [35, 40, 52], burning sensation [32, 41, 47, 52], taste alteration [41, 47] and dry mouth [47] etc. Consequently, the duration of CHX mouthwash application was mainly limited to four weeks in the included studies. Side effects of CPC mouthwash have been reported, including unpleasant flavor, burning sensation, taste alteration, and dry mouth, with a similar incidence to CHX [47]. Herbal mouthwashes also have side effects, with relatively low incidence compared to CHX [47, 52] and CPC [47] mouthwash. Many studies did not clearly state any side effects, which compromised the safety of mouthwashes. Orthodontic treatment is a long-term process with an average treatment time of 24.9 months [74]. However, apart from fluoride mouthwashes, the follow-up time of other mouthwashes was generally fewer than three months, leading to insufficient data to assess their efficacy and safety. Thus, the duration of mouthwashes used should be considered with caution.

Similar systematic reviews have been conducted regarding the efficacy of mouthwashes in orthodontic patients. For instance, it had been reported that Aloe Vera mouthwash is comparable to CHX in relieving gingival inflammation but not as effective for reducing dental plaque [75]. And the anti-gingivitis and anti-microbial effects of herbal mouthwashes compared to CHX were inconclusive [76]. However, the effect of multiple types of mouthwash on microorganisms has not been adequately evaluated.

In the current study, we critically reviewed the efficacy of multiple types of mouthwash on microorganisms and gingivitis compared to placebos or blank controls in orthodontic patients. In addition to the databases, we also searched a range of grey literature. Limitations in the current review remain. For instance, there are relatively wide variations regarding participants’ characteristics, prescription of mouthwashes, and other clinical and research methodologies, which may lead to heterogeneity. There were also methodological flaws in some of the included studies, such as small sample sizes and a high risk of bias, compromising the certainty of evidence.

The PICO question presented in this review has a positive answer. Proof supported the efficacy of mouthwashes in reducing oral microorganisms and/or relieving gingivitis in orthodontic patients. The efficacy was relevant to mouthwash types. Fluoride mouthwashes show short-term anti-microbial effects against *MS*, while the efficacy in relieving gingivitis was uncertain. CHX mouthwashes demonstrate efficacy in anti-multiple microorganisms and relieving gingivitis in the short-term. The efficacy of herbal mouthwashes was uncertain in anti-*MS,* while they have a relieving effect on gingivitis in the short-term. However, limited research has been conducted on the efficacy of other types of mouthwashes on oral microorganisms and gingivitis, requiring further research.

Future high-quality studies are needed. For instance, most studies assessed the level of specific microorganisms based on the cultivation methods or PCR. Only one study performed next-generation sequencing to detect the effect of mouthwash on the composition of the whole microbiota. Further studies with high-throughput and high-accuracy techniques are needed. Moreover, studies with more participants are encouraged to achieve the optimal information size. And it is essential to maintain a low loss to follow-up rate and high compliance during the trial to reduce the bias of missing outcome data.

## Conclusion

Based on the current findings, several mouthwashes demonstrated efficacy against oral microorganisms and/or in relieving gingivitis symptoms in orthodontic patients when compared to placebo or blank controls. Among that, fluoride mouthwashes appear to be effective in reducing colony counts of cariogenic bacteria in the short-term, while its long-term effect remains inconclusive. The efficacy of fluoride mouthwashes in reducing plaque- and gingival inflammation-related index is also uncertain. Short-term application of CHX may be effective in reducing multiple microorganisms. CHX and herbal mouthwashes effectively reduce gingival inflammation-related index, and there is also considerable agreement for plaque related index among included studies. The research on other types of mouthwashes is relatively limited and requiring further research. However, due to the certainty of evidence being very low or low for most comparisons, firm conclusions cannot be drawn. Further high-quality RCTs are still needed.

## Supplementary Information

**Additional file 1**: **Table S1.** Details for the reports excluded.

**Additional file 2**: **Table S2.** Relevant characteristics of included studies.

**Additional file 3**: **Table S3.** Funding sources of the included studies.

**Additional file 4**: **Figure S1.** Sensitive analysis. (a) Gingival Index by Löe and Silness (Chlorhexidine mouthwashes versus placebos), (b) Gingival Index by Löe and Silness (Chlorhexidine mouthwashes versus blank controls), (c) Plaque Index by Silness and Löe (Chlorhexidine mouthwashes versus blank controls), (d) Gingival Index by Löe and Silness (Herbal mouthwashes versus blank controls).

**Additional file 5.** PRISMA 2020 for Abstracts checklist.

## Data Availability

The datasets supporting the conclusions are included in the article and its additional files.

## Abbreviations

WMDs: Weighted mean differences
95%CIs: 95% Confidence intervals
*S. mutans*: S*treptococcus mutans*
*LB*: *Lactobacilli*
*P. gingivalis*: *Porphyromonas gingivalis*
CHX: Chlorhexidine
CPC: Cetylpyridinium chloride
PRISMA: Preferred reporting items for systematic reviews and meta-analyses
PICOS: Participant-intervention-comparison-outcomes-study design
RCTs: Randomized controlled trials
CENTRAL: Cochrane central register of controlled trials
ICTRP: International clinical trials registry platform
CPCI-S: The conference proceedings citation index-science
PQDT: The proquest dissertations and theses
PCR: Polymerase chain reaction
SD: Standard deviation
*I*^*2*^: I squared statistic
PI: Plaque index by Silness and Löe
GI: Gingival index by Löe and Silness
*MS*: *Mutans streptococci*
*T. forsythia*: *Tannerella forsythia*
*T. denticola*: *Treponema denticola*
*C. albicans*: *Candida albicans*
PD: Pocket probing depth
