# Supplementary material for: The efficacy of mouthwashes on oral microorganisms and gingivitis in patients undergoing orthodontic treatment: a systematic review and meta-analysis
Source: BMC Oral Health. 2023 Apr 6;23:204. doi: 10.1186/s12903-023-02920-4 (PMC10077628; doi:10.1186/s12903-023-02920-4)
Supplement: Supplementary file 1 — Additional file 1: Table S1. Details for the reports excluded. [file 12903_2023_2920_MOESM1_ESM.docx]

| **Details for the reports not retrieved** | | |
| --- | --- | --- |
| Reason | Studies | No. of studies |
| Full text not available | Amitha 1995 [1], Pontier 1990 [2], De Santis 2018 [3], Babay 1996 [4]. | 4 |
| **Details for the reports excluded after reading full text with reasons** | | |
| Reason | Studies | No. of studies |
| Wrong intervention(s) or  comparison(s) | Jurišić 2018 [5], Ramaglia 1999 [6], Jackson 1991 [7], Erverdi 2001 [8], Dhingra 2011 [9], Menezes 2006 [10], Erbe 2019 [11], Dogan 2008 [12], Lundström 1987 [13], Bagatin 2017 [14], Morrow 1992 [15], Gehlen 2000-1 [16], Gehlen 2000-2 [17], Nelson-Filho 2012 [18], Panhóca 2016 [19], Cosola 2019 [20], Lundström 1979 [21], Rose 2020 [22]. | 18 |
| Not RCT | Tufekci 2008 [23], Akbulut 2020-1 [24], Boyd 1989 [25], Shahana 2016 [26], Schaefer 2010 [27], Jurišić 2016 [28], Dogan 2009 [29], Sueishi 2021 [30], Chitra 2022 [31]. | 9 |
| *In-vitro* studies | Lessa 2007 [32], Nelson-Filho 2011 [33], Farhadifard 2021 [34], | 3 |
| Letter | Ballini 2019 [35]. | 1 |
| Wrong outcome(s) | Akbulut 2020-2 [36] | 1 |

**Additional file 1. Table S1. Details for the reports excluded**

**Reference:**

1. Amitha H, Munshi AK: Effect of chlorhexidine gluconate mouth wash on the plaque microflora in children using intra oral appliances. The Journal of clinical pediatric dentistry 1995, 20(1):23-29.

2. Pontier JP, Pine C, Jackson DL, DiDonato AK, Close J, Moore PA: Efficacy of a prebrushing rinse for orthodontic patients. Clinical preventive dentistry 1990, 12(3):12-17.

3. De Santis D, Pancera P, Luciano U, Gelpi F, Causarano G, Formentini D, Marchiori M, Lanaro L, Puddu G, Sinigaglia S *et al*: Evaluation of bacterial flora composition on teeth and periodontal tissues in patients in treatment with rapid palatal expander. Journal of biological regulators and homeostatic agents 2018, 32(2 Suppl. 2):31-36.

4. Babay N, Al Jasser N: Subgingival irrigation effects of chlorhexidine or sanguinarine on gingivitis in orthodontic patients. The Journal of clinical pediatric dentistry 1996, 20(3):225-228.

5. Jurišić S, Verzak Ž, Jurišić G, Jurić H: Assessment of efficacy of two chlorhexidine mouthrinses on oral hygiene and gingival health in adolescents wearing two types of orthodontic brackets. International journal of dental hygiene 2018, 16(2):e52-e57.

6. Ramaglia L, Sbordone L, Ciaglia RN, Barone A, Martina R: A clinical comparison of the efficacy and efficiency of two professional prophylaxis procedures in orthodontic patients. European journal of orthodontics 1999, 21(4):423-428.

7. Jackson CL: Comparison between electric toothbrushing and manual toothbrushing, with and without oral irrigation, for oral hygiene of orthodontic patients. American journal of orthodontics and dentofacial orthopedics : official publication of the American Association of Orthodontists, its constituent societies, and the American Board of Orthodontics 1991, 99(1):15-20.

8. Erverdi N, Acar A, Işgüden B, Kadir T: Investigation of bacteremia after orthodontic banding and debanding following chlorhexidine mouth wash application. The Angle orthodontist 2001, 71(3):190-194.

9. Dhingra K, Vandana KL: Management of gingival inflammation in orthodontic patients with ozonated water irrigation--a pilot study. International journal of dental hygiene 2011, 9(4):296-302.

10. Menezes SM, Cordeiro LN, Viana GS: Punica granatum (pomegranate) extract is active against dental plaque. Journal of herbal pharmacotherapy 2006, 6(2):79-92.

11. Erbe C, Klukowska M, Timm HC, Barker ML, van der Wielen J, Wehrbein H: A randomized controlled trial of a power brush/irrigator/mouthrinse routine on plaque and gingivitis reduction in orthodontic patients. The Angle orthodontist 2019, 89(3):378-384.

12. Dogan AA, Adiloglu AK, Onal S, Cetin ES, Polat E, Uskun E, Koksal F: Short-term relative antibacterial effect of octenidine dihydrochloride on the oral microflora in orthodontically treated patients. International Journal of Infectious Diseases 2008, 12(6):e19-e25.

13. Lundström F, Krasse B: Streptococcus mutans and lactobacilli frequency in orthodontic patients; the effect of chlorhexidine treatments. European journal of orthodontics 1987, 9(2):109-116.

14. Bagatin CR, Andrucioli MCD, Ferreira JTL, Matsumoto MAN, da Silva RAB, da Silva LAB, Romano FL, Nelson-Filho P: Biofilm formation in Haas palatal expanders with and without use of an antimicrobial agent: an in situ study. Microscopy research and technique 2017, 80(5):471-477.

15. Morrow D, Wood DP, Speechley M: Clinical effect of subgingival chlorhexidine irrigation on gingivitis in adolescent orthodontic patients. American journal of orthodontics and dentofacial orthopedics : official publication of the American Association of Orthodontists, its constituent societies, and the American Board of Orthodontics 1992, 101(5):408-413.

16. Gehlen I, Netuschil L, Berg R, Reich E, Katsaros C: The influence of a 0.2% chlorhexidine mouthrinse on plaque regrowth in orthodontic patients. A randomized prospective study. Part I: clinical parameters. Journal of orofacial orthopedics = Fortschritte der Kieferorthopadie : Organ/official journal Deutsche Gesellschaft fur Kieferorthopadie 2000, 61(1):54-62.

17. Gehlen I, Netuschil L, Georg T, Reich E, Berg R, Katsaros C: The influence of a 0.2% chlorhexidine mouthrinse on plaque regrowth in orthodontic patients. A randomized prospective study. Part II: Bacteriological parameters. Journal of orofacial orthopedics = Fortschritte der Kieferorthopadie : Organ/official journal Deutsche Gesellschaft fur Kieferorthopadie 2000, 61(2):138-148.

18. Nelson-Filho P, Carpio-Horta KO, Andrucioli MC, Feres M, Bezerra da Silva RA, Garcia Paula-Silva FW, Romano FL: Molecular detection of Aggregatibacter actinomycetemcomitans on metallic brackets by the checkerboard DNA-DNA hybridization technique. American journal of orthodontics and dentofacial orthopedics : official publication of the American Association of Orthodontists, its constituent societies, and the American Board of Orthodontics 2012, 142(4):481-486.

19. Panhóca VH, Esteban Florez FL, Corrêa TQ, Paolillo FR, de Souza CW, Bagnato VS: Oral Decontamination of Orthodontic Patients Using Photodynamic Therapy Mediated by Blue-Light Irradiation and Curcumin Associated with Sodium Dodecyl Sulfate. Photomedicine and laser surgery 2016, 34(9):411-417.

20. Cosola S, Giammarinaro E, Genovesi AM, Pisante R, Poli G, Covani U, Marconcini S: A short-term study of the effects of ozone irrigation in an orthodontic population with fixed appliances. European journal of paediatric dentistry 2019, 20(1):15-18.

21. Lundström F, Hamp SE, Nyman S: Systematic plaque control in children undergoing long-term orthodontic treatment. European journal of orthodontics 1979, 2(1):27-39.

22. Rose J, Ghoneima A, Lippert F, Maxwell L, Eckert G, Stewart KT: A visual evaluation of oral plaque removal utilizing an adjunct enzyme pre-rinse in orthodontic subjects. The Angle orthodontist 2020, 90(6):844-850.

23. Tufekci E, Casagrande ZA, Lindauer SJ, Fowler CE, Williams KT: Effectiveness of an essential oil mouthrinse in improving oral health in orthodontic patients. The Angle orthodontist 2008, 78(2):294-298.

24. Akbulut Y: The effects of different antiseptic mouthwash on microbiota around orthodontic mini-screw. Nigerian journal of clinical practice 2020, 23(11):1507-1513.

25. Boyd RL: Effects on gingivitis of daily rinsing with 1.5% H2O2. Journal of clinical periodontology 1989, 16(9):557-562.

26. Shahana RY, Muralidharan NP: Efficacy of mouth rinse in maintaining oral health of patients attending orthodontic clinics. Research Journal of Pharmacy and Technology 2016, 9(11):1991-1993.

27. Schaefer I, Braumann B: Halitosis, oral health and quality of life during treatment with Invisalign(®) and the effect of a low-dose chlorhexidine solution. Journal of orofacial orthopedics = Fortschritte der Kieferorthopadie : Organ/official journal Deutsche Gesellschaft fur Kieferorthopadie 2010, 71(6):430-441.

28. Jurišić S, Kozomara D, Jurić H, Verzak Ž, Jurišić G: The influence of different types of brackets and efficacy of two chlorhexidine mouthwashes on oral hygiene and the incidence of white spot lesions in adolescents during the orthodontic therapy. Psychiatria Danubina 2016, 28 Suppl 2:247-252.

29. Dogan AA, Cetin ES, Hüssein E, Adiloglu AK: Microbiological evaluation of octenidine dihydrochloride mouth rinse after 5 days' use in orthodontic patients. The Angle orthodontist 2009, 79(4):766-772.

30. Sueishi N, Ohshima T, Oikawa T, Takemura H, Kasai M, Kitano K, Maeda N, Nakamura Y: Plaque-removal effect of ultrafine bubble water: Oral application in patients undergoing orthodontic treatment. Dental materials journal 2021, 40(2):272-278.

31. Chitra P, Prashantha GS, Rao A: In vivo investigation of gingival health and oxidative stress changes in patients undergoing orthodontic treatment with and without fluoride use. J Indian Soc Periodontol 2022, 26(2):123-129.

32. Lessa FC, Enoki C, Ito IY, Faria G, Matsumoto MA, Nelson-Filho P: In-vivo evaluation of the bacterial contamination and disinfection of acrylic baseplates of removable orthodontic appliances. American journal of orthodontics and dentofacial orthopedics : official publication of the American Association of Orthodontists, its constituent societies, and the American Board of Orthodontics 2007, 131(6):705.e711-707.

33. Nelson-Filho P, Olmedo LY, Andrucioli MC, Saraiva Mda C, Matsumoto MA, de Queiroz AM, da Silva RA, da Silva LA: Use of the checkerboard DNA-DNA hybridisation technique for in vivo detection of cariogenic microorganisms on metallic brackets, with or without use of an antimicrobial agent. Journal of dentistry 2011, 39(7):513-517.

34. Farhadifard H, Soheilifar S, Bakhshaei A: Plaque Removal Efficacy of Three Cleaning Methods for Removable Orthodontic Appliances: A Crossover Randomized Clinical Trial. Turk J Orthod 2021, 34(3):170-175.

35. Ballini A, Cantore S, Fotopoulou EA, Georgakopoulos IP, Athanasiou E, Bellos D, Paduanelli G, Saini R, Dipalma G, Inchingolo F: Combined sea salt-based oral rinse with Xylitol in orthodontic patients: clinical and microbiological study. Journal of biological regulators and homeostatic agents 2019, 33(1):263-268.

36. Akbulut Y: The effect of different mouthwashes on bacteremia after debonding. Nigerian journal of clinical practice 2020, 23(7):900-905.
