# Supplementary material for: The efficacy of mouthwashes on oral microorganisms and gingivitis in patients undergoing orthodontic treatment: a systematic review and meta-analysis
Source: BMC Oral Health. 2023 Apr 6;23:204. doi: 10.1186/s12903-023-02920-4 (PMC10077628; doi:10.1186/s12903-023-02920-4)
Supplement: Supplementary file 2 — Additional file 2: Table S2. Relevant characteristics of included studies. [file 12903_2023_2920_MOESM2_ESM.docx]

**Additional file 2: Table S2. Relevant characteristics of included studies**

| Study | Sample  (gender) | Age  range | Mouthwash prescription | | Comparison | | Clinical measure | Effect | Follow  up | Loss  (%) | Side  effect |
| --- | --- | --- | --- | --- | --- | --- | --- | --- | --- | --- | --- |
| Alves 2010. | 30  (20F, 10M) | 12-21 | G1: Listerine ® (containing essential oils), 20 ml, 30 s, twice a day. | | G2: routine orientation | G3:  placebo,  20 ml,  30 s, twice a day. | PI, GI. | Compared to G2, significantly reduced GI was found in G1; Significantly reduced PI were found in G3 and G1. Compared to G3, insignificantly reduced PI and significantly reduced vestibular GI were found in G1. | 60  days | NR | No |
| Anderson  1997. | 32* | 11-15 | G1: Peridex ® (containing 0.12 % CHX), 15 ml, 30 s, twice a day after breakfast and before bedtime. | | G2: flavored placebo solution | | PI, GI, PD. | Compared to G2, significantly reduced PI and GI were found in G1; Significant reduced PD was found in G1 except mid-lingual site. | 3 months | 4 (1) | Burning sensation of mucosa, tooth stain. |
| Brightman  1991. | 36  (21F, 15M) | 11-17 | G1: Peridex ® (containing 0.12 % CHX), 1/2 oz, 30 s, twice a day. | | G2: placebo mouthwash, 1/2 oz, 30 s, twice a day. | | PI, GI, EIBI. | Significantly reduced PI, GI, and EIBI were found in G1 compared to G2. | 12  weeks | 2  (6) | Tooth stain |
| Chen  2013. | 90  (47F, 43M) | ≥ 13 | G1: Listerine ® (containing essential oils), 20 ml, 30 s, twice a day. | G2: fructus mume mouthwash (two-stage): rinse with 20 ml solution A for 30 s following rinse with 20 ml solution B for 30 s, twice a day. | G3: blank control (oral hygiene instruction alone) | | TI, MGI, BI, Log_10_CFU/ml of total aerobic and anaerobic bacteria, *Streptococci*, and *LB*. | Compared to G3, significantly reduced BI was found in G1 and G2. No signiﬁcant intergroup differences in total anaerobic and aerobic bacteria, *Streptococci*, *LB* counts were found between three groups. | 6 months | 11 (12) | NR |

Continued.

| Study | Sample  (gender) | Age  range | Mouthwash prescription | | | | Comparison | Clinical measure | Effect | Follow  up | Loss  (%) | Side  effect |
| --- | --- | --- | --- | --- | --- | --- | --- | --- | --- | --- | --- | --- |
| Dadgar  2021. | 38  (24F, 14M) | 12-30 | G1: 0.05% fluoride mouthwash, 5 ml, twice a day. | | G2: probiotic mouthwash contained 10^8^ CFU of Lactobacillus,  5 ml, twice a day. | | G3: placebo mouthwash, 5 ml, twice a day. | Colony count of *MS* in dental plaque | There was no significant difference in *MS* among the three groups. The *MS* increased in G3 and significantly reduced in G1 compared to baseline. There was no significant change over time in G2. | 2  weeks | NR | NR |
| Dehghani  2015. | 60  (27M, 33F) | 15-22 | G1: mouthwash containing 0.06 % CHX and 0.05 % NaF,15 ml,  1 min, twice a day. | G2: 0.06 % CHX,15 ml, 1 min, twice a day. | | G3: 0.05 % NaF, 15 ml, 1 min, twice a day. | G4: placebo, 15 ml,  1 min, twice a day. | MGI, BI, TI, Log_10_CFU/ml of total bacterial, *MS* and *LB* in dental plaque. | Compared to G4, significantly reduced MGI, BI, and TI were found in G1 and G2, significantly reduced BI was found in G3; Significantly reduced total bacteria and *MS* were found in G1, G2, and G3; Significantly reduced *LB* was found in G1. Compared to baseline, significantly reduced MGI, BI, TI, *MS* and *LB* were found in G3. | 21 days | NR | No |
| Dehghani  2019. | 37  (10M, 27F) | 15-35 | G1: mouthwash containing Propolis aqueous extract, twice a day, 15 ml,  1 min. | | G2: 0.12 % CHX, twice a day, 15 ml,  1 min. | | Before-after comparison | PI, GI, CPI. | Compared to baseline, significantly reduced PI, GI, and CPI were found in groups, with no significant difference between them. | 3  weeks | 3  (8) | G2: spicy taste, undesirable feeling, tooth stain. |

Continued.

| Study | Sample  (gender) | Age  range | Mouthwash prescription | | | | Comparison | | Clinical measure | Effect | Follow  up | Loss  (%) | Side  effect |
| --- | --- | --- | --- | --- | --- | --- | --- | --- | --- | --- | --- | --- | --- |
| Du  2004. | 48  (25M, 23F) | 10-17 | G1: oral hygiene instruction and 0.12 % CHX | | G2: oral hygiene instruction  and normal saline mouthwash | | G3: normal saline mouthwash | | Percentage of cocci, bacilli, spirochetes in dental plaque. | The G1 had better microbiologic change than G2 and G3. | 3 months | NR | NR |
| Enerbäck  2018. | 270  (90M,180F) | 12-20 | G1: 1450 ppm fluoride toothpaste plus 0.2 % Flux® NaF mouthwash, 2 min, 10 ml, twice a day. | | | | G2: 5000 ppm fluoride toothpaste | G3: 1450 ppm fluoride toothpaste | CFU (logarithmic mean) of *MS* and *LB* in saliva, PI. | Compared to baseline, significant increases in *MS* and *LB* were found in all groups. There were no significant differences in PI, *MS,* and *LB* between groups. | 1  year | 7  (3) | No |
| Fard  2001. | 25  (5M, 20F) | Mean: 19 ± 6/3 | G1: Ortho-kin® (containing CHX, NaF), 15 ml, 30 s, twice a day. | G2: Listerine ® (containing essential oils), 15 ml,  30 s, twice a day. | | G3: Oral-B® (containing CPC, NaF), 15 ml, 30 s, twice a day. | Before-after comparison | | *MS* scores in plaque and saliva, amount of dental plaque. | Compared to baseline, significantly reduced plaque was found in the three groups. Compared to G2 and G3, insignificantly reduced plaque amount and *MS* scores were found in G1. | 3 weeks +3 weeks interval +3 weeks | NR | NR |
| Farhadian  2015. | 72  (17M, 55F) | Mean:18.6 ± 4.8 | G1: Manual toothbrush and Poursina® Persica mouthwash, 15 ml, 20 s, twice a day. | | G2: Manual toothbrush and Shahre Daru® 0.2 % CHX,15 ml, 30 s, twice a day. | | G3: Manual toothbrush | G4: Electric toothbrush | BOP, GI, PI’. | Compared to baseline, significantly reduced BOP, GI, and PI’ were found in all groups. Compared to G3, significantly reduced BOP in G1 was found. | 2  weeks | NR | NR |

Continued.

| Study | Sample  (gender) | Age  range | Mouthwash prescription | | | Comparison | Clinical measure | Effect | Follow  up | Loss  (%) | Side  effect |
| --- | --- | --- | --- | --- | --- | --- | --- | --- | --- | --- | --- |
| Faria  2020. | 31  (17F, 14M) | 12-35 | G1: 0.5 % Zingiber officinale essential oil mouthwash,  10 ml, 60 s, once daily. | G2: 0.12 % CHX,  10 ml, 60 s, once daily. | | G3: flavored sterile placebo, 10 ml, 60 s, once daily. | BBI, BI’, Log_10_CFU/ml of *MS* and *C. albicans* in saliva. | Compared to baseline, significantly reduced BBI were found in all groups; Significantly reduced *MS* was found in G2. Compared to G3, significantly reduced BI’ was found in G1 and G2; Significantly reduced *MS* was found in G1 and G2. No signiﬁcant change of *C. albicans* was found. | 7 days +15 days interval +7 days | NR | Unpleasant  flavor |
| Goes  2016. | 30  (4M, 26F) | 10-40 | G1: 0.12 % CHX,  15 ml, 1 min, twice a day. | | G2: 1 % MTC mouthwash, 15 ml,  1 min, twice a day. | G3: placebo, 15 ml,  1 min, twice a day. | VPI, GBI. | A significant reduction in VPI and GBI was found in G1 and G2 compared to G3. | 15 days | 0  (0) | Burning or taste change in G1, tongue numbness in G3. |
| Goyal  2019. | 45* | 15-35 | G1: probiotic mouthwash, 15 ml, 60 s, twice a day. | | G2: 1000 ppm  amine fluoride mouthwash, 15 ml, 60 s, twice a day. | G3: blank control (regular cleansing aids) | *P. gingivalis* level in plaque sample (RT‑PCR) | Compared to baseline, significantly reduced *P. gingivalis* level was found in G1; Insignificant increase in *P. gingivalis* levels was found in G2. | 6  months | NR | No side effects in G1, brown staining inG2. |
| Hasriati  2020. | 30  (25F, 5M) | 15-33 | G1: KITOBE®1 % chitosan with 0.25 % acetic acid, 10 ml, 30 s, twice a day. | G2: MINOSEP® 0.2 % CHX,  10 ml, 30 s, twice a day. | | G3: sterile aquadest,  10 ml, 30 s, twice a day. | Total bacteria (CFU/ml) and red complex bacteria count (qPCR) | Significantly reduced total bacteria were found in G1 and G2 compared to G3, with no significant difference between them. Three groups exhibited reduced red-complex bacteria count. | 4 days | 0 (0) | No |

Continued.

| Study | Sample  (gender) | Age  range | Mouthwash prescription | | Comparison | Clinical measure | Effect | Follow  up | Loss  (%) | Side  effect |
| --- | --- | --- | --- | --- | --- | --- | --- | --- | --- | --- |
| Koopman  2015. | 120* | 10-16.8 | G1: Elmex® mouthwash containing 100 ppm AmF and 150 ppm NaF | | G2: placebo | 16S rRNA sequencing of dental plaque. | The effects of the mouthwash on the microbial composition were minor. | 28  months | 22  (18) | NR |
| Lin  2014. | 45  (22M, 23F) | 16-30 | G1: Listerine® mouthwash (containing essential oils), 10 ml, 60 s, twice a day. | G2: tap water, 10 ml, 60 s, twice a day. | G3: blank control (toothbrush, four times daily) | SBI, TI, MGI’. | In addition to MGI’, Significant reduced SBI and TI were found in G1 compared to baseline. | 1 month | NR | NR |
| Madlena  2012. | 40  (14M,26F) | Mean: 20.1 ± 5.6 | G1: Meridol® AmF/SnF_2_ mouthwash, 10 ml, 30 s, twice a day, and AmF/SnF_2_ toothpaste, 3min, twice a day. | | G2: 1400 ppm fluoride AmF/SnF_2_ toothpaste, 3 min, twice a day. | BOP, GI, PI. | Compared to baseline, BOP, GI, and PI were significantly reduced in the two groups, with no significant difference between them. | 4  weeks | 1  (3) | No |
| Maruo  2008. | 64  (19M, 32F) * | 7-11.5 | G1: 0.2 % CHX, 5 ml, 30 s, three times a day. | | G2: no antimicrobial treatment. | Biofilm mass (mg) and CFU/mg of total *Streptococci* biofilm. | Compared to G2, total *Streptococci* counts in G1 were significantly reduced. There was no significant difference in biofilm mass between groups. | 7  days | 13  (20) | Yellow, brown-stained teeth in G1, mucosal desquamation |

Continued.

| Study | Sample  (gender) | Age  range | Mouthwash prescription | | | | Comparison | | Clinical measure | Effect | Follow  up | Loss  (%) | Side  effect |
| --- | --- | --- | --- | --- | --- | --- | --- | --- | --- | --- | --- | --- | --- |
| Niazi  2018. | 100  (79F, 21M) | 13-37 | G1: 0.2 % CHX, 10 ml, 1 min, twice a day. | G2: 0.05 % CPC,  10 ml,  1 min, twice a  day. | G3:10 % Salvadora persica,  10 ml, 1 min, twice a day. | G4: 10 % Azadirachta indica,  10 ml, 1 min, twice a day. | Blank control (toothpaste and toothbrush) | | BBPI | Compared to blank control, significantly reduced BBPI were found in four groups. Compared to G1, significantly reduced BBPI was found in G3. | 3 weeks (blank control) +3  weeks (mouthwash) | 15 (15) | Taste alteration, burning sensation, dry mouth, unpleasant taste. |
| Nishad  2017. | 60* | 18-35 | G1: CHX, 5 ml, twice a day. | | G2: Azadirachta indica mouthwash, 5 ml, twice a day. | | G3: distilled water,  5 ml, twice a day. | | PI’’, GI’, *MS* colony count (CFU/ml) in saliva. | Compared to baseline, significantly reduced *MS* colony count were found in G1 and G2. Compared to G2 and G3, significantly reduced PI’’, GI’, and *MS* was found in G1. | 30  days | NR | Tooth stain |
| Ousehal  2011. | 84  (21M,63F) | mean: 19.41 | G1: manual toothbrush combined with Kin® mouthwash with a 0.12 % concentration of CHX and 0 % alcohol. | | | | G2: manual toothbrush | G3: electric toothbrush | GI, PI. | Compared to G2, insignificantly reduced PI was found in G1; Significantly reduced GI was found in G1. | 4  weeks | NR | NR |
| Pahwa  2011. | 45  (20M,25F) | 11-25 | G1: Crest® 0.07 % CPC mouthwash, 10 ml, 60 s, twice a day. | | | | G2: toothpaste and toothbrush | G3: placebo, 10 ml,  60 s, twice a day. | MGI, PI, GBI. | Compared to baseline, significantly reduced GBI was found in G1; No significant changes of MGI were found. Compared to G2, significantly reduced PI was found in G1. | 1  month | NR | NR |

Continued.

| Study | Sample  (gender) | Age  range | Mouthwash prescription | | | | Comparison | | Clinical measure | Effect | Follow  up | Loss  (%) | Side  effect |
| --- | --- | --- | --- | --- | --- | --- | --- | --- | --- | --- | --- | --- | --- |
| Saffari  2015. | 30* | 13-17 | G1: Behsa® 0.2 % CHX,  15 ml, 30 s, twice a day. | | G2: Poursina® Persica, 15 ml, 30 s, twice a day. | | Before-after comparison | | CFU of *MS colonies* in elastic O-rings. | Compared to baseline, significantly reduced *MS* was found in G1; Insignificant reduced *MS* was found in G2. | 4  weeks | NR | NR |
| Salehi  2006. | 60* | 13-18 | G1: Poursina® Persica, 15 ml, 20 s, twice a day. | | G2: Shahr Daru® 0.2 % CHX,  15 ml, 30 s, twice a day. | | G3: water, 15 ml, 30 s, twice a day. | | CFU of *MS colonies* in elastic ring. | Compared to G3, significantly reduced *MS* was found in G1 and G2. G2 was significantly better than G1. | 20  days | NR | Tooth stain, unpleasant taste, burning sensation. |
| Shah  2019. | 30* | NR | G1: 0.2 % CHX,  20 ml, twice a day. | | G2: probiotic mouthwash with  2 ×10^8^ CFU/g of microorganisms, twice a day. | | G3: blank control | | PI, GI, CFU/ml of *MS* in saliva. | Compared to G3, significantly reduced PI, GI, and the *MS* count was found in G1 and G2. | 28  days | NR | NR |
| Shalini  2018. | 32* | 12-30 | G1: 0.2 % CHX, 10 ml, 1 min, twice a day. | G2: herbal mouthwash 10 ml,  1 min, twice a day. | | G3: green tea extract mouthwash, 10 ml, 1min, twice a day. | Before-after comparison | | PI’’ | Compared to baseline, significantly reduced PI’’ was found in three groups. Compared to G3, significantly reduced PI’’ was found in G2 and G1. | 14  days | NR | No |
| Shilpa  2019. | 111* | 13-35 | G1: manual toothbrush combined with Hexidine® 0.2 % CHX. | | | | G2: manual toothbrush | G3: powered toothbrush | PI, GI, MPBI | Compared to G2, significantly reduced PI, GI, and MPBI were found in G1. | 2  months | NR | NR |

Continued.

| Study | Sample  (gender) | Age  range | Mouthwash prescription | | | | Comparison | Clinical measure | Effect | Follow  up | Loss  (%) | Side  effect |
| --- | --- | --- | --- | --- | --- | --- | --- | --- | --- | --- | --- | --- |
| Sobouti  2018. | 54  (23M,31F) | 12-21 | G1: Poursina® persica, dilute 10 drops of the solution within 2 spoons of water, thrice a day. | | G2: Ortho-Kin® (containing diluted CHX and fluoride),15 ml, 30 s, twice a day. | | G3: placebo,15 ml, 30 s, twice a day. | PI’, GI, GBI’, PD | Compared to G3, significantly reduced PI’ was found in G1 and G2; significantly reduced GI and GBI’ were found in G1; significantly higher PD changes were found in G2. | 1  month | 5  (9) | No |
| Van der Kaaij  2015. | 120  (35M,85F) | 10-18 | G1: Elmex® mouthwash contained 250 ppm fluoride (100 ppm amine fluoride and 150 ppm sodium fluoride). | | | | G2: fluoride-free placebo | BS | Compared to G2, insignificantly reduced BS in G1was found. | 24.5 ± 5.5 months | 22  (18) | NR |
| Yeturu  2016. | 90  (40M, 45F) * | ＞18 | G1: aloe vera mouthwash 10 ml,  1 min, twice a day. | G2: CHX mouthwash, 10 ml,  1 min, twice a day. | | G3: mouthwash containing ClO_2_, 10 ml, 1 min, twice a day. | Before-after comparison | PI’’’, GI | Compared to baseline, significantly reduced PI’’’ and GI were found in three groups. Compared to G1, significantly reduced PI’’’ and GI were found in G2. | 15  days | 5  (5) | NR |
| Zingler  2016. | 48  (19F, 29M) | median: 12.5 (G1)  13.0 (G2) | G1: 100 ppm AmF/150 ppm NaF mouthwash, 10 ml, 30 s, twice a day. | | | | G2: blank control (toothbrush) | API, PBI, CFU/ml of *MS* and *LB* in saliva. | Changes in *MS* and *LB* levels, and PBI were not significant during the trial nor between groups. Compared to G2, slightly reduced API was found in G1 (significant at 6 and 9 months). | 12 months | 3 (6) | NR |

NR, not reported; *LB, Lactobacilli*; *MS, Mutans streptococci*; CFU, colony forming units; MTC, Matricaria chamomilla; CPC, cetylpyridinium chloride; ClO_2_: chlorine dioxide.

**Plaque-related indices:** PI, Plaque Index by Silness and Löe 1964; TI, Quigley-Hein Plaque Index; MS scores, colony-forming units of *Mutans Streptococcus*; PI’, Plaque Index by O’Leary’s 1972; BBI, Bonded Bracket Index; VPI, Visible Plaque index; BBPI, Bonded Bracket Plaque Index; PI’’, Plaque Index (Unspecified); PI’’’, Plaque Index by William 1991; API, Approximal Plaque Index.
**Gingival inflammation-related indices:** GI, Gingival Index by Löe and Silness 1963; PD, probing depth; EIBI, Eastman Interproximal Bleeding Index; MGI, Modified Gingival Index by Lobene 1986; BI, Bleeding Index by Saxton and van der Ouderaa 1989; BI’, Bleeding Index (Unspecified); CPI, Community Periodontal Index; BOP, bleeding on probing; GBI, Gingival Bleeding Index by Ainamo J 1975; BS, Bleeding Score; SBI, Sulcus Bleeding Index; MGI’, Modified Gingival Index (Unspecified); GI’, Gingival Index (Unspecified); MPBI, Modified Papillary Bleeding Index; GBI’, Gingival Bleeding Index by Carter and Barnes 1974; PBI, Papillary Bleeding Index.

*Full data not available
