# Supplementary material for: The efficacy of mouthwashes on oral microorganisms and gingivitis in patients undergoing orthodontic treatment: a systematic review and meta-analysis
Source: BMC Oral Health. 2023 Apr 6;23:204. doi: 10.1186/s12903-023-02920-4 (PMC10077628; doi:10.1186/s12903-023-02920-4)
Supplement: Supplementary file 3 — Additional file 3: Table S3. Funding sources of the included studies. [file 12903_2023_2920_MOESM3_ESM.docx]

| **Additional file 3: Table S3. Funding sources of the included studies** | |  |
| --- | --- | --- |
| Category | Study | No. of studies |
| Industry sponsored studies | Anderson 1997 was partially supported by Procter and Gamble (mouthwashes), no other source of funding indicated. Enerbäck 2018 received donations (mouthwashes, toothbrushes, and toothpaste) from Colgate-Palmolive (Lyngby, Denmark), Actavis (Stockholm, Sweden), and Lactona (Bergen op Zoom, Holland). Koopman 2015 was supported by Elmex research/Colgate-Palmolive Europe. Madlena 2012 received donations (study materials) from GABA International (Therwil, Switzerland), no other source of funding indicated. Van der Kaai 2015 was supported by Elmex research/Colgate-Palmolive Europe, Therwil, Switzerland. Zingler 2016 received donations (study materials) from GABA GmbH (Lörrach, Germany) and Ivoclar Vivadent AG (Schaan, Liechtenstein), no other source of funding indicated. | 6 |
| Non-industry sponsored studies  (Government or non-profit entity) | Chen 2013 was supported by the 2008 Innovation in Oral Care Awards (International Association for Dental Research/GlaxoSmithKline). Dehghani 2015 and Dehghani 2019 were supported by the Research Council of Mashhad University of Medical Sciences, Mashhad, Iran. Enerbäck 2018 was supported by Forskning och utveckling, Västra Götaland, Sweden. Farhadian 2015 was supported by Research Centre of Hamadan University of Medical Sciences. Goes 2016 was supported by grants from Brazilian agencies. Hasriati 2020 was supported by the Directorate of Research and Community Engagement, Universitas Indonesia. Maruo 2008 was supported by CAPES, Brazil. Saffari 2015 was supported by Shahid Sadoughi University of Medical Sciences. Salehi 2006 was supported by the office of Vice-Chancellor for Research of Shiraz University of Medical Sciences. Shah 2019 was supported by the Indian Council of Medical Research. | 11 |
| Other | Alves 2010, Du 2004, Brightman 1991, Fard 2001, Faria 2020, Lin 2014, Niazi 2018, Ousehal 2011, Pahwa 2011 and Yeturu 2016 did not disclose funding sources. Dadgar 2021, Nishad 2017, Shalini 2018, Goyal 2019, and Shilpa 2019 indicated no financial support and sponsorship. Sobouti 2018 was supported by the authors from Mazandaran university of medical sciences, Sari, Iran. | 16 |
