# Supplementary material for: The efficacy of mouthwashes on oral microorganisms and gingivitis in patients undergoing orthodontic treatment: a systematic review and meta-analysis
Source: BMC Oral Health. 2023 Apr 6;23:204. doi: 10.1186/s12903-023-02920-4 (PMC10077628; doi:10.1186/s12903-023-02920-4)
Supplement: Supplementary file 4 — Additional file 4: Figure S1. Sensitive analysis. (a) Gingival Index by Löe and Silness (Chlorhexidine mouthwashes versus placebos), (b) Gingival Index by Löe and Silness (Chlorhexidine mouthwashes versus blank controls), (c) Plaque Index by Silness and Löe (Chlorhexidine mouthwashes versus blank controls), (d) Gingival Index by Löe and Silness (Herbal mouthwashes versus blank controls). [file 12903_2023_2920_MOESM4_ESM.docx]

**Additional file 4: Figure S1. Sensitive analysis. (a)** Gingival Index by Löe and Silness (Chlorhexidine mouthwashes versus placebos), (b) Gingival Index by Löe and Silness (Chlorhexidine mouthwashes versus blank controls), (c) Plaque Index by Silness and Löe (Chlorhexidine mouthwashes versus blank controls), (d) Gingival Index by Löe and Silness (Herbal mouthwashes versus blank controls).
